# Plant Polyphenols and Exendin-4 Prevent Hyperactivity and TNF-α Release in LPS-Treated *In vitro* Neuron/Astrocyte/Microglial Networks

**DOI:** 10.3389/fnins.2017.00500

**Published:** 2017-09-06

**Authors:** Francesca Gullo, Michela Ceriani, Alessia D'Aloia, Enzo Wanke, Andrew Constanti, Barbara Costa, Marzia Lecchi

**Affiliations:** ^1^Department of Biotechnology and Biosciences and Milan Center for Neuroscience, University of Milano-Bicocca Milan, Italy; ^2^Department of Biotechnology and Biosciences, University of Milano-Bicocca Milan, Italy; ^3^Department of Pharmacology, School of Pharmacy, University College London London, United Kingdom

**Keywords:** sterile inflammation, LPS, TNF-α, GLP-1, plant polyphenols, exendin-4 (EX-4), neocortical cultures, multi-electrode array (MEA)

## Abstract

Increasing evidence supports a decisive role for neuroinflammation in the neurodegenerative process of several central nervous system (CNS) disorders. Microglia are essential mediators of neuroinflammation and can regulate a broad spectrum of cellular responses by releasing reactive oxygen intermediates, nitric oxide, proteases, excitatory amino acids, and cytokines. We have recently shown that also in *ex-vivo* cortical networks of neurons, astrocytes and microglia, an increased level of tumor necrosis factor-alpha (TNF-α) was detected a few hours after exposure to the bacterial endotoxin lipopolysaccharide (LPS). Simultaneously, an atypical “seizure-like” neuronal network activity was recorded by multi-electrode array (MEA) electrophysiology. These effects were prevented by minocycline, an established anti-inflammatory antibiotic. We show here that the same inhibitory effect against LPS-induced neuroinflammation is exerted also by natural plant compounds, polyphenols, such as curcumin (CU, curcuma longa), crocin (CR, saffron), and resveratrol (RE, grape), as well as by the glucagon like peptide-1 receptor (GLP-1R) agonist exendin-4 (EX-4). The drugs tested also caused *per-se* early transient (variable) changes of network activity. Since it has been reported that LPS-induced neuroinflammation causes rearrangements of glutamate transporters in astrocytes and microglia, we suggest that neural activity could be putatively increased by an imbalance of glial glutamate transporter activity, leading to prolonged synaptic glutamatergic dysregulation.

## Introduction

Microglia are an integral part of central nervous system (CNS) networks, forming the innate defensive system, and their pathological potential has been extensively investigated (Kettenmann et al., 2011). In different pathologies, microglia acquire distinct functional states and, during the disease progression, modify and change their activated phenotype. Activated microglia specifically interact with neurons and influence their survival either in a positive or in a negative direction. They can physically contact injured neurons and remove synapses, a process termed synaptic stripping (Kettenmann et al., 2013). Conceptually, microglial cells not only can affect neural networks through removal of cellular and subcellular elements and secreting cytokines, trophic factors, and neurotransmitters (Kettenmann et al., 2011), but also by receiving messages; in fact, they express a variety of receptors for neurotransmitters, neuropeptides, and neuromodulators. Thus they have also the capacity to sense neuronal activity (Pocock and Kettenmann, 2007).

Current pharmacological interventions against neuroinflammation have symptomatic benefits to certain degrees, but do not prevent progressive neurodegeneration. More recently, it has been shown that some natural substances protect the substantia nigra dopaminergic neurons (involved in the onset of Parkinson's disease) through their anti-inflammatory action. Among these are some polyphenols such as curcumin, CU (Ojha et al., 2012), resveratrol, RE (Zhang et al., 2010; Gao et al., 2014; Lofrumento et al., 2014), and peptide molecules such as exendin-4, EX-4 (Kim et al., 2009; Athauda and Foltynie, 2016). Moreover, also crocin, CR, extracted from saffron, resulted in an effective protection from macular degeneration, a retinal disease principally related to oxidative stress and chronic inflammation (Nam et al., 2010; Marangoni et al., 2013). In many of these reports, sterile inflammation was induced, microglia-released cytokine concentration increased, but the properties of neuronal network activity were never explored.

By using low concentrations of the purified bacterial endotoxin lipopolysaccharide (LPS) over 6–8 h, we reproduced *in vitro* a “sterile” CNS neuroinflammation in a 10,000-cell network of neurons, astrocytes and microglia, grown on a multielectrode array (MEA) dish, where neurons were regularly bursting for weeks (Gullo et al., 2014). We found that an “atypical” neuronal excitability took place causing long-lasting bursts resembling epileptiform seizures. These slow changes of neuronal excitability were accompanied by a simultaneous increase in microglia-released tumor necrosis factor-alpha (TNF-α) concentration, suggesting a crucial role of microglia, but not of astrocytes, in this process. Both these effects were blocked by pre-treatment with minocycline, an anti-inflammatory antibiotic drug, which was inactive when applied alone.

Here, we wished to examine whether the action of minocycline on our neuron/astrocyte/microglial co-culture system could be reproduced by the above mentioned natural molecules as anti-inflammatory tools. By simultaneously using an electrophysiological recording technique, such as microelectrode arrays (MEA), and TNF-α ELISA assays, we found that all of these molecules, depending on the concentration used, were transiently able to modify the balanced network activity and to imitate the blocking effects of minocycline against LPS neuroinflammation. Furthermore, all of the agents tested resulted in an anti-inflammatory action at concentrations lower than those generally reported in the literature.

## Materials and methods

### Cell cultures

Primary cultures of cortical neurons were prepared from post-natal mice (P1–P3) as previously described (Gullo et al., 2009). All the procedures concerning animal handling and sacrifice followed the Principles of Laboratory Animal Care (2010/63/UE Directive), and were approved by the University of Milan-Bicocca Ethics Committee and the Italian Ministry of Health (D.Lgs 26/2014). Briefly, the cerebral cortex (excluding the hippocampus) was removed from decapitated mice, cut into 1 mm^3^ pieces, and digested by trypsin (0.15%) and DNAse (10 μg/ml) at 37°C for 20 min. After enzyme digestion, cells were mechanically dissociated by means of trituration, and plated at densities of 600–900 × 10^3^ cells/ml on glass coverslips (for immunocytochemistry) or MEA Petri dishes pre-coated with polyethyleneimine 0.1% (wt/vol) and laminin 20 μg/ml (30 μm diameter ITO electrodes spaced 200 μm apart, Multichannels System, Germany). After 3 h incubation, the plating medium was replaced by neurobasal medium (NB) with B27 (Invitrogen, Italy), glutamine 1 mM and basic fibroblast growth factor (bFGF) 10 ng/ml, and the culture was maintained at 37°C in 5% CO_2_. One-half of the medium volume was replaced every 3 days. The cultures in MEA dishes were covered with gas-permeable covers (MEA-MEM, Ala Scientific Instruments, Inc., USA) throughout the culture period (12–22 days-*in-vitro*, DIV).

### MEA electrophysiology: drug application-general aspects

As previously described (Gullo et al., 2009), we report results obtained within a few hours after positioning the MEA dish into the incubator, which can be considered at the steady-state. The recording area in our MEA dishes was ~2 mm^2^, and in this area, the average number of neurons was in the order of ~5,000, plus about the same number of astrocytes (see Figure 1 of Supplementary Material in Gullo et al., 2010, at http://www/frontiersin.org/neuralcircuits/paper/10.3389/fncir.2010.00011/). The average space between cells was therefore relatively large. Neuroinflammation was induced by incubating cultures with LPS, isolated and purified from *E. coli* R515 by Enzo Life Sciences (Alexis Biochemicals, code 581-007). This product does not contain detectable protein or DNA contaminants with agonistic Toll-like receptor (TLR) activity. Since it is a strong activator of TLR4 but it does not activate TLR2 or the other TLRs, it was considered suitable for our experiments. The drugs to be tested were kept as frozen stock solutions in distilled water (or DMSO <0.1%) at −20°C, until diluted as appropriate with MEA culture medium before each experiment. The drugs used were: crocin (CR), the polyphenols resveratrol (RE) and curcumin (CU), purchased from Sigma, Italy; exendin-4 (EX-4) and the GLP-1 receptor antagonist, exendin (9–39), purchased from Tocris, UK. All experiments were performed by adding the drugs in volumes that were always <1% of the total conditioned media volume bathing the neurons. When indicated, a washout was carried out with a solution pre-conditioned by the same network under control conditions.

### MEA electrophysiology: recordings, waveform acquisition, and sorting

We used the same procedures previously described in Gullo et al. (2009, 2010). Briefly, analog signals sampled at 40 kHz were recorded at 36°C in CO_2_-controlled incubators using MEA-1060BC or 1060INV pre-amplifiers (bandwidth 1–8,000 Hz, Multichannel Systems, Germany) connected to a MEA Workstation (bandwidth 100–8,000 Hz, Plexon Inc., USA). Data were sorted into timestamp files by the MEAWorkstation Sorter software (MEAWS, see details below) and cleaned of artifacts using the OFFLine Sorter program (Plexon Inc., USA). Next, during the PCA-based waveform sorting and for multi-unit electrodes, we applied one of the following procedures: (i) spike removal with a Mahalanobis threshold in the range 1.8 to 1.4; we concurrently checked that the *P*-value of multivariate ANOVA sorting quality statistics was <0.01 among the identified units; (ii) when the previous procedure led to excessive spike invalidation, we manually removed the spikes invading the adjacent unit ellipsoids (the latter method was very effective in decreasing the *P*-values, with only a limited number of erased spikes).

### Neuronal cluster classification

The method of classification into excitatory or inhibitory neurons is described in detail in Gullo et al. (2009, 2010) and Becchetti et al. (2012). Briefly, for each identified unit and each burst, the following characteristics were computed in defined time segments: the autocorrelation function (ACF), the burst duration (BD), the spike number (SN), the spike rate (SR), the intra-burst spike rate (IBSR), the inter-burst intervals (IBIs), and the Fano factor (FF; Teich, 1989; Baddeley et al., 1997). We classified the neurons on the basis of an unsupervised learning approach consisting of data reducing principal component analysis (PCA) based on FF as a feature (Becchetti et al., 2012), followed by the K-means clustering procedure. The large differences in these burst metrics was the basis for adopting FF as the best feature to clusterize neurons. As previously described (Becchetti et al., 2012), these procedures normally separated two statistically different clusters composed of numbers of excitatory (~60–90) and inhibitory (~15–25) neurons, whose ratio always fitted the ratio present in the neocortex i.e., from 4 to 5 (Gullo et al., 2010; Sahara et al., 2012). To give an approximate idea of the computing time involved in the analysis of a 60-electrode MEA dish with ~100 identified neurons, the following are the typical parameters: 20 h-long experiment, 0.5 GB memory size, 10 min to obtain results.

### Advanced tools for characterizing firing and BD histograms

**Figure 1:**
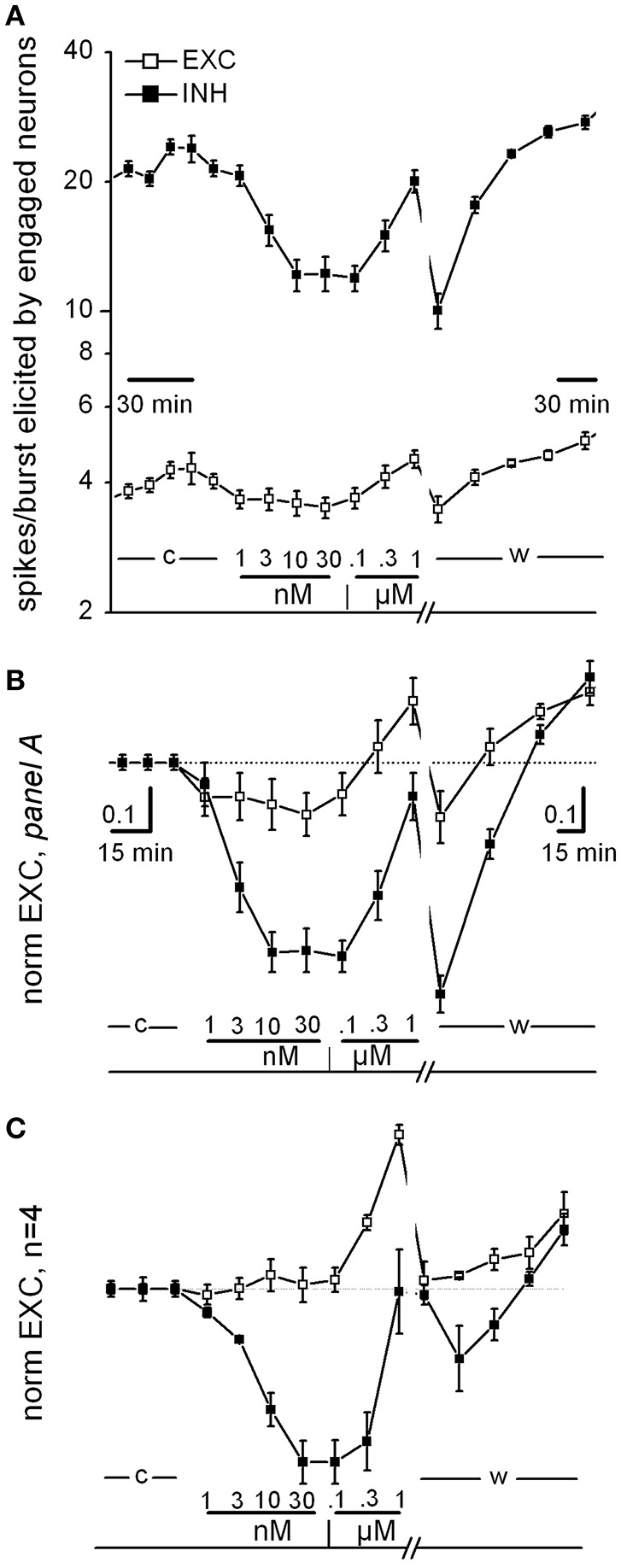
Curcumin dose-response relationship. **(A)** Time plot of excitability (EXC) for inhibitory and excitatory neurons (closed and open symbols, respectively) recorded in one exemplary dish. Data before and after the washout time break (13 min) have different time resolution (see calibration bars). The time course of curcumin (CU) application is described above the bottom line as follows: c, w, control and washout; numbers are [CU] in nM and μM as indicated. **(B)** Same data as in **(A)**, but values were normalized to those of control and superimposed in order to be compared. Note that in this experiment, CU either decreased the excitability of both inhibitory and excitatory cells in the 1–30 nM concentration region or increased excitability in the further 30–1,000 nM concentration region, in a reversible manner. **(C)** Plot as in **(B)**, but average of data from four independent experiments from different dishes but similar DIVs.

The global network burst structure was analyzed with standard techniques as well as procedures recently developed by us (Gullo et al., 2012). Briefly, we applied a running window of variable duration (from 10 ms to 1 s) in order to search for the start and end of each burst and simultaneously collecting all of the spikes which were precisely tagged to the engaged excitatory and inhibitory neurons, already designated as previously explained above. In conclusion, for each spike in a generic burst, we knew exactly which neuron fired it and how many other spikes were fired by all of the other neurons. This had the consequence that we could also compute for the two identified neuronal clusters (defined above) the average propensity for firing, namely, the average number of spikes per burst, here called “excitability” (EXC) in each time segment of interest (shown in Figure 1A). Normally, it is much more interesting to compare EXC data normalized to control for both clusters, namely, normEXC. To investigate the heterogeneity of the burst length, BD, we studied its distribution in the form of cumulative probabilities in each time segment of interest, cumBD, as previously done in Gullo et al. (2014). To compare experiments, two types of plots are shown as follows: (i) time plots where *normEXC* describes how clusters change firing in the presence of drugs with respect to control (open, □, and closed, ■, symbols for excitatory and inhibitory clusters, respectively). Aligned characters, attached to the time axis, are control (c), washout (w), or drug names (short names were: CU, CR, RE, EX-4) indicating the presence or absence of drugs (note axis breaks necessary to zoom to particular time segments); (ii) *cumBD* plots (bin 0.2 s) vs. burst duration describing how drugs modify the distribution of the burst lifetimes (thick continuous lines for control; open stars for drugs; open rightward triangles, ⊳, for LPS and open upward triangles, Δ, for washout. In our previous paper (Gullo et al., 2014), we found that, at 6–7 h after LPS application, its action was either present or blocked if the values of two variables, namely normEXC and cumBD_95_ (the value of BD at cumBD of 95%), resulted in an increase with respect to control beside fixed thresholds. The normEXC threshold was +45 ± 10% (with respect to control value, in this case 1) and cumBD threshold was +40 ± 5% (with respect to the BD value in control computed at cumBD_95_). Therefore, to highlight if drugs were able or unable to block LPS action in the normEXC and cumBD_95_ plots, in the figures these thresholds were represented by a thin straight line and an arrow respectively.

### TNF-α concentration measurements

In each MEA dish, small (150 μl) aliquots of the incubation medium were collected in control and during different times (6 and 12 h, representing the peak-time of LPS-induced TNF-α release, Gullo et al., 2014) after the addition of LPS (3 μg/ml) or polyphenols/crocin/exendin-4 + LPS. Samples (50 μl) were analyzed in triplicate for murine TNF-α with enzyme-linked immunosorbent assay (ELISA) kits (KCM3012, Invitrogen, Italy) according to manufacturer's instructions. The data were expressed as pg/ml following interpolation on the basis of a standard curve. As suggested by the manufacturer, the inter-assay and intra-assay variability were 3.5–4.3 and 2.6–8.2%, with a lower limit of detection <3 pg/ml. During the analysis we did not correct the data for the small change in the total incubation medium volume resulting from the aliquots removal. Data were analyzed using GraphPad 7.0 software employing ANOVA followed by Dunnet's test for group comparison. *P* < 0.05 was considered statistically significant.

### Data analysis and statistical significance

All of the data are expressed as mean values ± SEM, with *n* indicating the number of experiments. We used OriginPro 9.1 to test normality of data such as cumBD and FSH. Moreover, for statistical significance among cumBD and FSH distributions from different experiments, we used the non-parametric tests Wilcoxon signed rank test or Kruskal–Wallis test both available from OriginPro 9.1. The data were analyzed and the figures prepared using OriginPro 7.0 or OriginPro 9.1 software.

## Results

To study the activity of reverberating cortical networks, we followed the procedures described in Gullo et al. (2014). In our cultures, neurons, astrocytes and microglial cells survived together for weeks. In these conditions, neurons were able to generate spikes, which were stably recorded by MEA system (60 recording electrodes, 200 μm apart). The spikes were acquired during each burst (every ~10 s) consisting of an ~1 s-brief period of synaptic-mediated global network activity among all excitable cells. Indeed, each elicited spike was the result of the temporal and spatial summation of a minimum of ~100 postsynaptic potentials reaching each of the ~3,500 neurons present in the dish. On the whole, each burst was a reverberating event of the spontaneous network activity consisting of a total of ~600 spikes elicited by neurons (~75 and ~25 excitatory and inhibitory cells, at ~4 and 12/burst, respectively). This means that data recorded from a single burst were the outcome of ~60,000 synaptic events, but it should be kept in mind that this was a significant (*n* = ~100 electrodes) sample compared to the ~3,500 interconnected neurons present in the whole network.

When the culture dishes reached maturation (from 10 up to 22 DIV) we performed experiments, lasting more than ~15 h, during which no significant changes in network activity were observed under control conditions. These cultures were therefore useful for acquiring data in the long-lasting test on the LPS effects, in which we recorded the atypical “seizure-like” activity (Gullo et al., 2014). To study the neuronal activity, we analyzed firing data by using two variables fully describing the temporal changes: (a) plots of normEXC (excitability normalized to control) of the clusters of excitatory and inhibitory neurons and (b) plots of cumBD (cumulative distribution of the burst durations). Since we found that the drugs were able to induce relatively fast, but transient effects (compared to control data and long-lasting LPS effects, see Gullo et al., 2014), we analyzed results in graphs whose time axis contained appropriate breaks and drug labels. Moreover, to decide if the LPS action should be considered successful or blocked by drugs, we fixed thresholds in normEXC and cumBD_95_ (the value of BD at cumBD of 95%) plots as a thin straight line and a vertical arrow, respectively (see Section Advanced Tools for Characterizing Firing and BD Histograms). Drugs were always tested, before applying LPS, by a brief preconditioning (30–60 min) interval in which a drug was added to the incubation medium.

### Are drugs intrinsically affecting excitability?

For each tested drug, we performed a dose-response curve using concentrations based on published data, and then we verified if the substances *per*-*se* introduced relevant excitability changes with respect to control and washout.

In Figure 1, we show some specific exemplary experiments for CU; similar experiments were done also for the other drugs. As shown in Figure 1A (an exemplary experiment out of 4 similar results), the CU dose-response relationships consistently confirmed that in the low nanomolar region, both excitatory and inhibitory neurons decreased their activity immediately (over a few minute time course). On the contrary, in the region from 30 nM up to 1 μM the CU effect was opposite. Finally, the effects were almost completely reversible on washout. This plot shows the time course of the average spikes/burst elicited by neurons engaged in the activity (“excitability,” EXC, see details in Section Methods) by the two inhibitory (in control ~20) and excitatory (~4.5) neuronal clusters. EXC is characterized by values that are very different between the clusters and can be changed by pharmacological manipulations (see Becchetti et al., 2012). The neuronal clusters responded immediately and differently after each drug application (10 min) starting from 1 nM up to 1 μM (see legend).

To compare different experiments, it was necessary to normalize data to the control data, and in Figures 1B,C these plot-types (now of “normEXC”) are shown superimposed. It can be noticed that from the response in B, the IC_50_ for inhibitory neurons (closed square) was ~3 nM with a saturation after 10 nM and a recovery during washout. In Figure 1C we show the average of 4 similar experiments and the normEXC dose-response curves were found to have two different shapes in the two concentration regions from 1 to 30 nM and from 0.1 to 1 μM, respectively. In the first region only inhibitory cells were inhibited, and the IC_50_ was 4.5 ± 0.6 nM; on the contrary, in the second region, inhibitory cells recovered their activity and excitatory cells increased their activity. On average, at 1 μM, the global effect was an increase of activity that recovered on washout. It has previously been shown that an early and fast effect of CU, characterized by an IC_50_ in the micromolar range, consists of inhibiting glutamate (glu) release from synaptosomes from rat cortex nerve endings (Lin et al., 2011, 2012).

On the whole, these results are only partially in line with those obtained by Lin et al. (2011) by using a different technique. Indeed, since in the cortex microcircuits, inhibitory neurons receive glutamatergic synaptic inputs (in both feedback and feedforward loops, see Isaacson and Scanziani, 2011), we expected to observe a consistent decrease in their activity only in the lower CU concentration region. Compared to those of Lin et al. (2011) (glutamate release from synaptosomes), our methods were characterized by the functional synapse integrity and the astrocyte-mediated [glu]_o_ uptake (Wanke et al., 2016), suggesting a much higher sensitivity. Moreover, our dose-response curves cannot be considered at steady-state because each drug concentration was changed every 10 min.

### Transient and long term effects of CU: LPS-induced inflammation is blocked at 1 μM CU

Since our experiments with LPS-induced inflammation imply very long time-segments of the order of about 18 h, we investigated if the inhibitory CU effects seen in Figure 1 were indeed transient and therefore eventually negligible over this time-frame. Moreover, we wanted to use CU concentrations lower than those that have been recently used in animals (~100 μg/Kg/day, see Ojha et al., 2012; Hoppe et al., 2013; Zhu et al., 2014) approximatively equivalent to ~100 μM. Indeed, since we did not have any blockade induced by the blood brain barrier (see Wang et al., 2016) and the drug action should be directly on neurons, we decided to fix 1 μM as a reasonable concentration, which is in line with the cited experiments performed by Lin et al. (2011).

**Figure 2:**
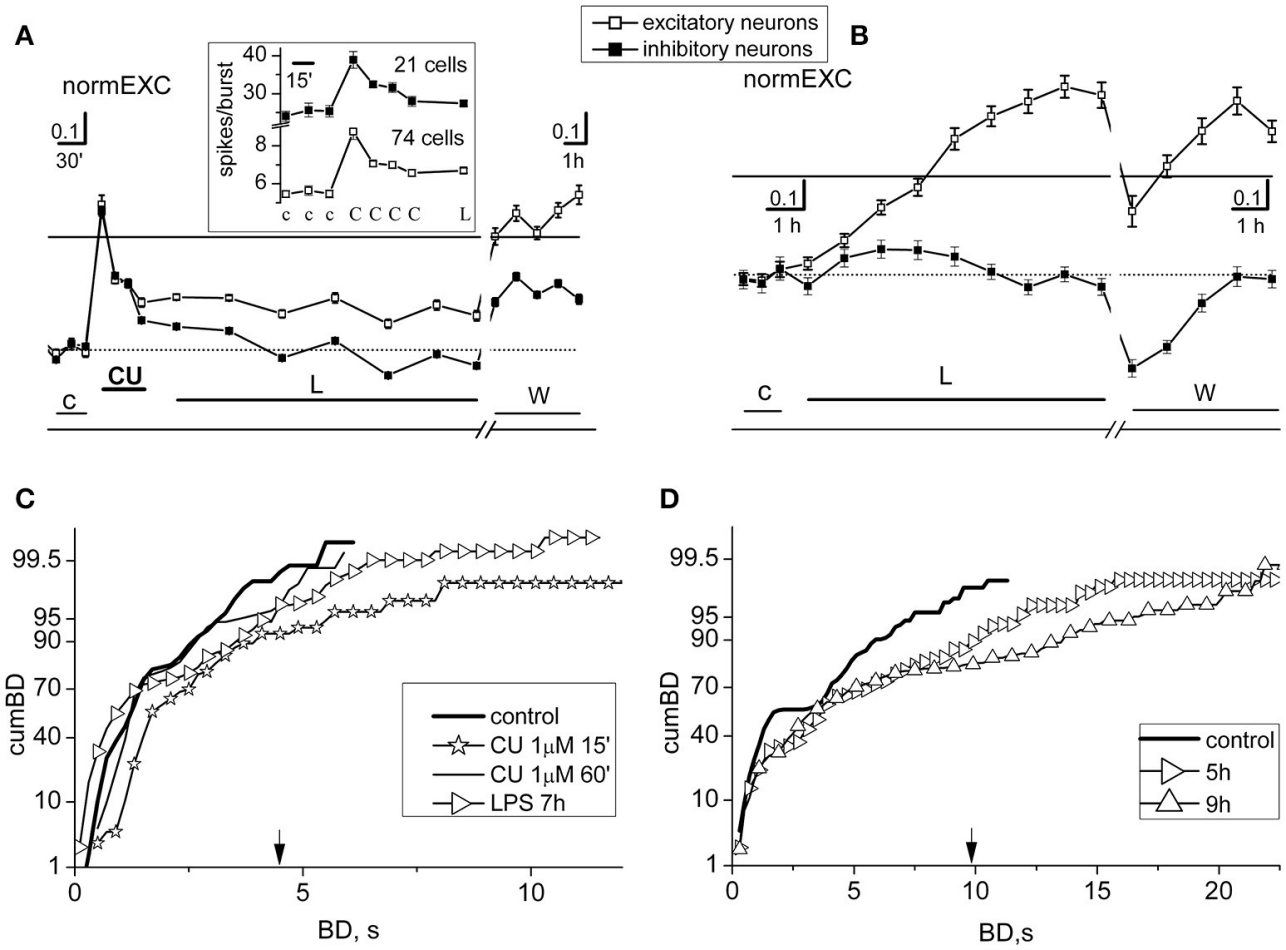
Short- and long-term effects of curcumin in networks treated with LPS. Results from two different experiments, performed in the same dish, with preconditioning or not-preconditioning with 1 μM curcumin (CU) (done 3 DIV later) before the application of LPS (3 μg/ml) are shown in **(A,C)** and **(B,D)**, respectively. **(A)** Plots of normEXC in control (c), 1 μM CU (CU), LPS (L), and during washout (w). Inset: for the brief (~3 h) initial time segment in control, CU and L, plot of average number of spikes/burst observed in neurons of both clusters. The continuous line is the fixed threshold. Other details: the total number of spikes from excitatory (inhibitory, in parenthesis) neurons in control and CU (in bold): 304.2 ± 18, **568.5** ± **32** (481.9 ± 33, **764.7** ± **45.4**); the total number of engaged excitatory (inhibitory, in parenthesis) neurons was in control: 52 ± 1.8, **63.8** ± **1.1** (17.8 ± 0.3, **19.3** ± **0.2**). **(B)** In the same dish as in **(A)**, but 3 DIV later, plots of normEXC in control (c), LPS (L) and during washout (w). The continuous line is the fixed threshold. **(C)** Plots of cumBD in different time regions of the experiment shown in **(A)**. Data of cumBD are superimposed as explained in text. **(D)** Plots of superimposed cumBD curves in different time regions of the experiment shown in **(B)**. Note that pre-conditioning with CU prevented the late, prolonged excitatory response to LPS. The vertical arrow indicates the fixed threshold.

Experiments were done by sequentially recording activity in control (3 h), during a preconditioning in CU (about 1 h at 1 μM) and successively in LPS (at least 7 h at 3 μg/ml); a final washout of 5 h was also recorded. The fast effects of a single drug application were studied in detail by averaging data every 15 min. After 3 days, in the same dish we performed a standard experiment by applying only LPS to be sure that the effects of the induced inflammation were still working. The results of such a dual experiment are shown in Figures 2A–D, respectively. In the experiment of Figure 2A that plots normEXC, 1 μM CU produced a fast and transient activity increase which actually decayed in ~45 min. In the inset are shown the original data before normalization: we specifically analyzed the average number of spikes elicited in each burst and in each cluster of neurons; this analysis was performed counting for each neuron the spikes engaged in each burst. In the first 15 min, the drug caused an immediate firing increase of the inhibitory and excitatory clusters from 25.4 ± 1.5 to 38.9 ± 2.2 and from 5.5 ± 0.2 to 8.73 ± 0.4 spikes/burst (the bursts were 82, number of inhibitory and excitatory cells, 21 and 74, respectively) (see other details in legend). The subsequent LPS application, in fact, caused a further *decline* of normEXC for both clusters of neurons and the trend of normEXC for excitatory and inhibitory clusters was +14 ± 3 and +9 ± 1% respectively, suggesting that LPS was unable to exert its expected excitatory inflammatory function as previously observed (Gullo et al., 2014). Indeed, as shown in Figure 2B, the effects of a standard LPS application (3 μg/ml), done in the same dish 3 DIV later, illustrate that, after 4 and up to 8 h, a strong increase of the activity of excitatory neurons (+86 ± 6%) took place, although we did not observe any effect on inhibitory cells.

The cumBD data associated with the Figures 2A,B experiments are shown in Figures 2C,D, respectively. In C four curves are superimposed as follows: (i) control (thick line), (ii) CU at 15 min (line + asterisk), (iii) CU at 60 min (thin line), (iv) LPS at 7 h (line + right-triangles). The corresponding 90% BD values were: 2.6, 3.8, 2.6, 3.7 s, respectively. These results suggest that, as compared to control, CU only transiently increased cumBD_90%_, and LPS over 7 h produced only a 37% increase, confirming that the expected LPS action did not take place. Statistical significance by Kruskal–Wallis test applied to control data and CU at 60 min resulted in a *P* < 0.12, and at 7 h in LPS data resulted in *P* < 0.05. In contrast, as shown in Figure 2D, a typical effect of LPS on cumBD (in the same dish) was to further right-shift, with respect to control (line, 95% at 7.2 s), the curves at 5 h (right-triangles) and at 9 h (upward-triangles) to 12 s (+66%) and 17 s (+136%) values, respectively, with “seizure-like” burst durations reaching 22 s. The Kruskal–Wallis test of significance applied to control data and LPS data resulted in *P*-values which were always smaller than 10^−4^.

On the whole, these results demonstrate that 1 μM CU by itself suddenly produced changes of activity in bursting, by promoting long BD values and significant increases in the excitability of both neuronal clusters. These transient modifications rapidly returned to quasi-control values as if the effects were partially compensated, only producing scarce or null effects in the early hours after adding LPS. On all of the occasions (*n* = 10) in which we performed these types of experiments, the results were similar. In other experiments (*n* = 5) in which we used smaller CU concentrations (i.e., 0.1 μM), the results were not clear and these data were discarded. Few (*n* = 4) experiments performed in dishes whose LPS responses were not significant were also discarded.

### Transient and long term effects of CR: LPS-induced inflammation is blocked at 20 μM CR

Crocin and crocetin have been found to block the release of NO and various chemokines under the action of LPS (Nam et al., 2010). As for curcumin, we did fast dose-response experiments initially to test putative effects of crocin (CR) on the activity of our networks (Supplementary Figure 1). In the range 1–100 μM, some inhibitory effects were seen only beyond 20 μM (*n* = 3). We here show that during the first hour of incubation, experiments using CR at 3 (*n* = 3) and 20 μM (*n* = 3) had either negligible or transient inhibitory effects, respectively. These CR effects were accompanied by other different actions caused by the further LPS presence.

**Figure 3:**
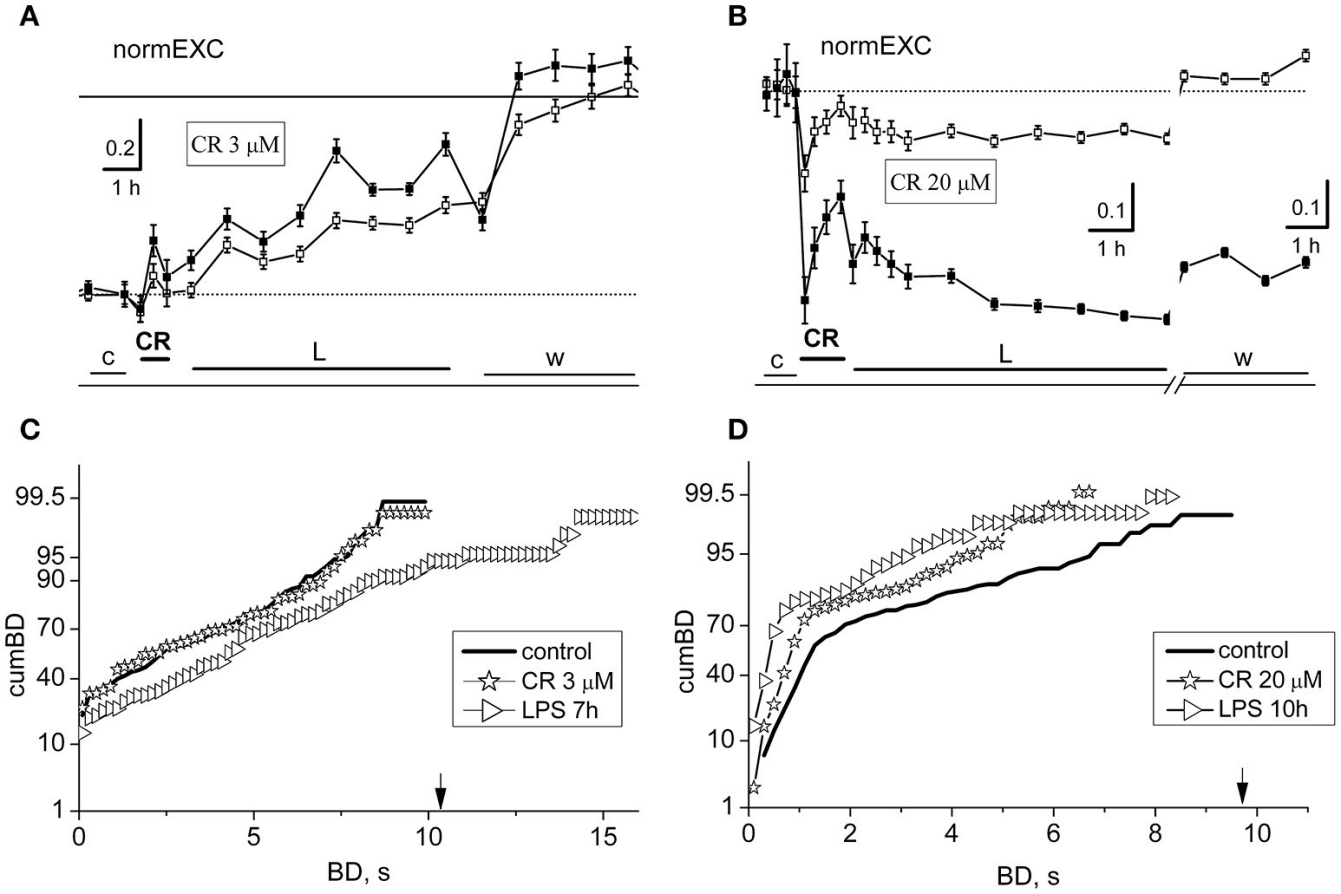
Short- and long-term effects of crocin (CR) in networks treated with LPS. Data in **(A,C)** and those in **(B,D)** belong to the same exemplary experiment. **(A,B)** Plots of normEXC data for two exemplary experiments in which the dishes were treated with crocin (CR) at 3 and 20 μM, respectively. Open and closed symbols indicate excitatory and inhibitory neurons. The continuous line is the fixed threshold. **(C)** Plot of cumBD in control (thick line), in the presence of 3 μM CR (asterisks), and during the seventh hour after the addition of LPS (3 μg/ml; right triangles). These two last curves were significantly different from the control curve with *P* < 0.05. **(D)** Plot of cumBD in control (thick line), in the presence of 20 μM CR (asterisks), and during the 10th h after the addition of LPS (right triangles). The vertical arrow indicates the fixed threshold. These last curves were significantly different from the control curve with *P* < 0.05. The highest dose of CR prevented the late excitatory LPS responses.

In Figure 3 are shown the results from two exemplary experiments at 3 (Figures 3A,C) and 20 (Figures 3B,D) μM CR, respectively. At 3 μM it can be observed in Figure 3A that the early CR-induced activity was barely modified and LPS induced a very slow, but sizeable increase of activity up to ~52% at eighth hours. An analysis to test the cumBD plots, shown in Figure 3C, suggests that a significant increase by 46% of BD was present at 7 h LPS as compared to control.

On the contrary, at 20 μM CR, the effects were found to be largely depressive. After the CR application (see Figure 3B), we detected an early negative peak of 45 and 16%, respectively in inhibitory and excitatory cells, but this effect decayed in the next hour. During the next 7 h in LPS, the activity remained stable but depressed. Furthermore, by analyzing in Figure 3D the cumBD plots, it can be concluded that the curves shifted to the left, suggesting that the burst durations decreased, in line with a weaker activity of the network.

On the whole, it can be concluded that CR is able to block the LPS-induced inflammation only at concentrations higher than 15–20 μM. Interestingly, at these concentrations, although the fast earlier transient inhibitory effects were present, a strong general inhibition of activity remained and was washed out only during recovery.

### Transient and long term effects of RE: LPS-induced inflammation is blocked at 1 μM RE

**Figure 4:**
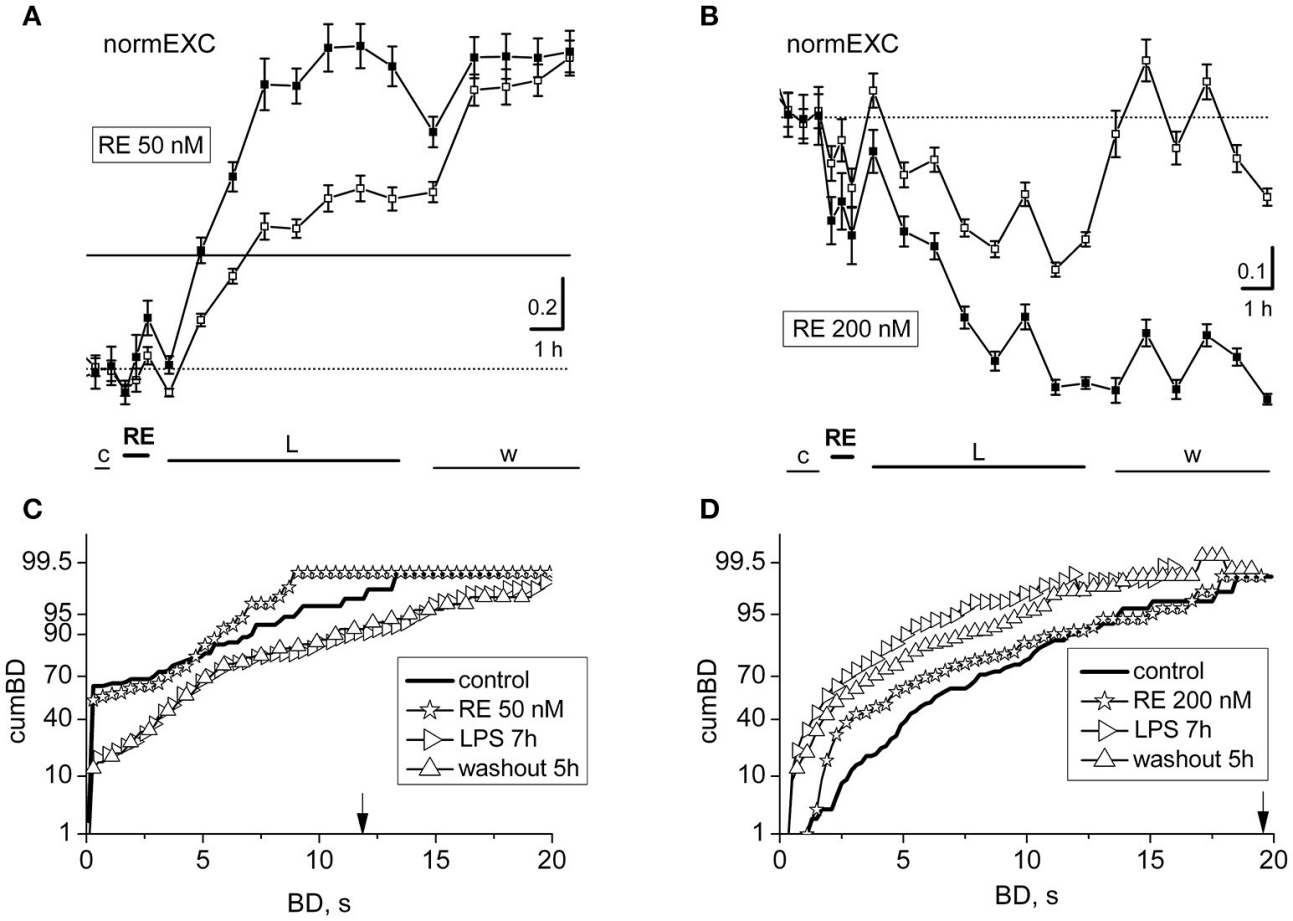
Effects of resveratrol (50 or 200 nM) on LPS responses. Data in **(A,B)** and **(C,D)** derive from two experiments performed on a single dish at 14 or 20 DIV. **(A,B)** Plots of normalized excitability following application of resveratrol (RE) 50 nM **(A)** or 200 nM **(B)** and LPS (3 μg/ml). The continuous line is the fixed threshold. Note that in **(A)**, the late excitatory responses to LPS were not prevented, whereas in **(B)** the responses were inhibited. **(C,D)** Plots of cumBD in the same conditions as in **(A,B)**. The vertical arrow indicates the fixed threshold.

We studied the action of resveratrol (RE) at concentrations in the lower micromolar range, much lower than those reported in many papers present in the literature (Gambini et al., 2015). As reported above for CU, also RE has been described to inhibit the release of glutamate from nerve terminals (Chang and Wang, 2009). These authors showed that the threshold to detect the start of the inhibition was between 10 and 100 nM, but at 1 μM the effect reached the IC_50_. When tested in our dishes, 200 nM RE (Figure 4B) exerted immediately a decrease in the neuronal firing, but the network slowly recovered after ~30 min.

Due to this reason, we decided to search for a concentration range in which effects were either unable or able to cause changes in the plots of normEXC and the cumBD in opposite directions. In Figures 4A,B are shown two exemplary experiments in which RE applied at 50 and 200 nM induced a negligible or a fast inhibitory effect, respectively, in the normEXC plots. The average duration of these intrinsic responses of RE were never longer than ~45 min. The subsequent application of LPS was tested every hour over 8 h.

In Figures 4A,B it can be observed that the development of the response was divergent, i.e.: normEXC data either increased in Figure 4A (at 50 nM) or decreased in Figure 4B (at 200 nM), respectively, suggesting that the drug preconditioning produced opposite results during the LPS action. Similar results were also seen in other 4 dishes at 50 nM and 3 dishes at 200 nM. The fluctuations of the activity shown in Figure 4A, during control and RE, were not significant up to the first hour of LPS application, whereas from 2 to 8 h in LPS, the activity increased as expected by 71% for excitatory cells and 127% for inhibitory cells (see in Gullo et al., 2014). On the other hand, in Figure 4B, the normEXC slowly decayed during the following 8 h.

To complete the analysis, we computed the distribution of the BD durations of these experiments and the results are shown in Figures 4C,D, respectively. Although the profile properties of the control curves (thick line) were different in the two experiments, the data acquired during the presence of RE alone show, in both cases, a cumBD curve (see the asterisk symbols) very similar to control. On the contrary, comparing the last hours of LPS (right-pointing triangles) data in Figure 4C suggest that the 90% cumBD showed an 87% increase, and in 4 D a dramatic decrease, thus confirming that the RE doses used in these experiments either did not block or completely blocked the LPS action, respectively.

### In agreement with data reported in hippocampal pyramidal neurons, EX-4 triggered fast transient pro-excitatory responses

In hippocampal pyramidal neurons *in vivo*, it has been shown that a brief (1 s) application of GLP-1 (7–36) amide, a naturally produced active fragment of GLP-1, first rapidly increased (i.e., 1 s) and then slowly decreased (>10 s) single unit firing activity (Oka et al., 1999). Interestingly, these effects were inhibited either by the specific GLP-1 receptor antagonist, exendin (9–39) or by the specific non-NMDA glutamate receptor antagonist 6-cyano-7-nitroquinoxaline-2,3-dione (CNQX), suggesting that the initial GLP-1 action involved a fast and transient release of glutamate.

**Figure 5:**
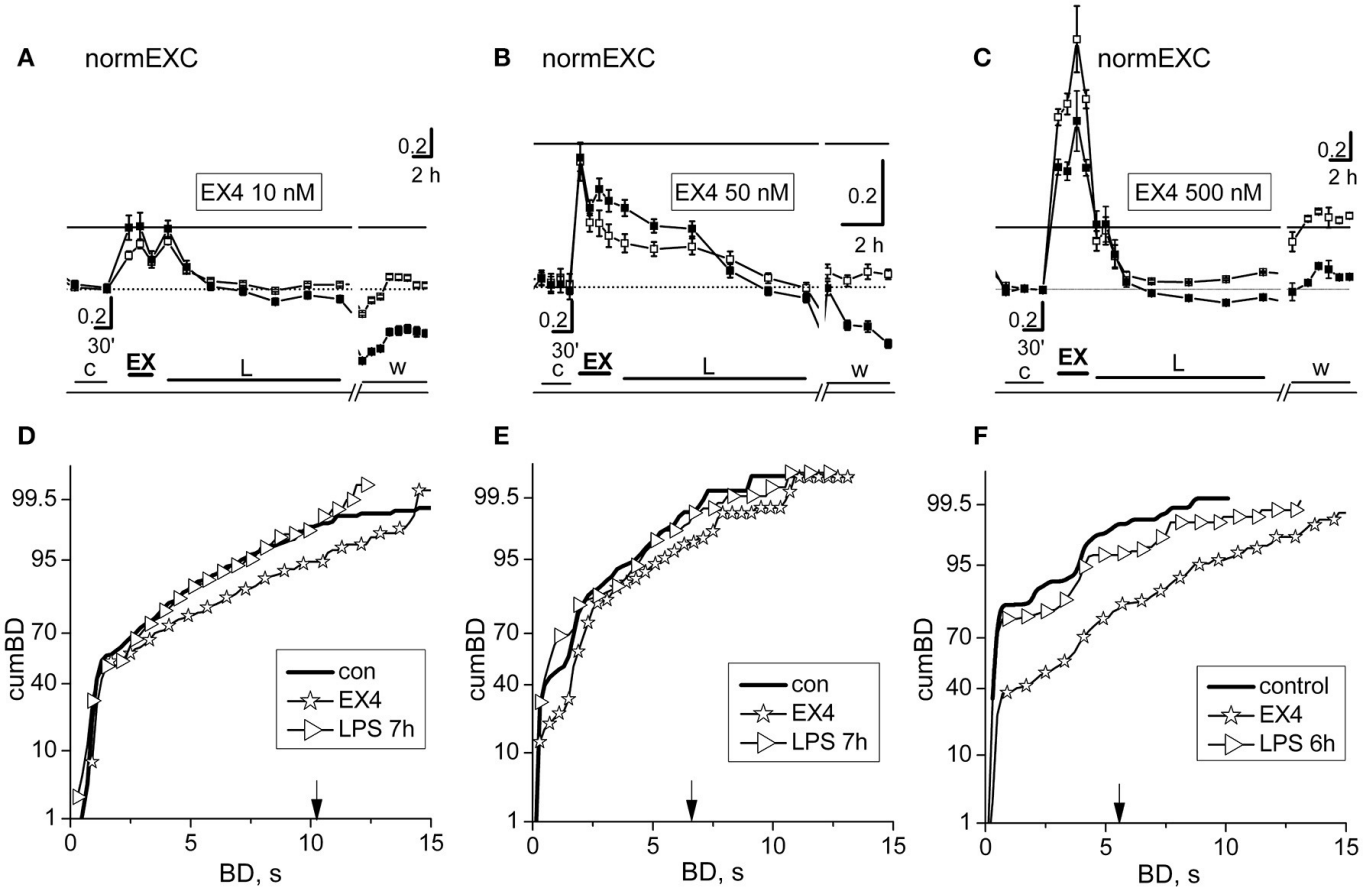
The fast and slow network responses to 10, 50, and 500 nM exendin-4 or exendin-4 + LPS. Data in **(A,D)** and **(C,F)** were obtained from the same dish in two different experiments performed 3 DIV apart. **(A–C)** NormEXC plot obtained with 10, 50, and 500 nM exendin-4 (EX-4) preconditioning, and 3 μg/ml LPS. The continuous line is the fixed threshold. Note that the transient and fast increase of normEXC became progressively higher with increasing EX-4 concentration. **(D–F)** CumBD plots of the experiments shown in **(A–C)**. Control, thick line; EX-4, asterisk; rightward triangles are for the late LPS effects. The vertical arrow indicates the fixed threshold. Note that preconditioning with EX-4 at each concentration tested, effectively prevented the development of the late excitatory responses to LPS.

Our *in vitro* networks of cortical neurons from neonatal mice contain fractions of excitatory and inhibitory cortical neurons, which always fit the standard ratio of 4:1 known to be present in adult cortex (Sahara et al., 2012). As for curcumin and crocin, we did fast dose-response experiments to test putative effects of EX-4 on the activity of our networks (Supplementary Figure 1). Surprisingly, we found that in about ~80% (*n* = 24) of the experiments done using EX-4, the fast effects consisted, as expected, of a transient, brief pro-excitability response, but there were also some networks (~20%, *n* = 6) whose responses consisted of a transient *decreased* excitability. This was probably caused by unknown heterogeneity factors intrinsic to our cultured preparation of dissociated cells, and therefore we excluded these experiments from the results (although they were characterized by late LPS responses where atypical activity was present). EX-4, from 10 up to 500 nM, elicited an early fast excitatory response and prevented the late LPS-induced neuroinflammation. Amongst the EX-4 experiments in which the early effects of the drug were pro-excitable, in Figure 5 are shown the results of two experiments, performed in the same dish (at two different DIVs) with 10 and 500 nM EX-4 and one experiment done at 50 nM. In Figures 5A–C are compared the normEXC data and it is evident that concentrations differing by 5 and 50 times indeed produced fast transient excitatory effects, suggesting a dose-dependent mechanism of action. On the contrary, the long-term normEXC curves at the end of the LPS application did not show any significant change, thus suggesting that activity of the network was not different with respect to control. We analyzed in Figures 5D–F the related cumBD histograms in control, during the early EX-4 action and at the end of the long-term LPS action. In the three cases, the early EX-4 histograms showed a conspicuous rightward shift of the curves. On the contrary, the final effect of LPS was either negligible or without atypical bursts showing a BD increase at 90% in the cumBD curves.

### The GLP-1 receptor antagonist exendin (9-39) blocked the fast transient EX-4 effect and the protective effect against LPS neuroinflammation

**Figure 6:**
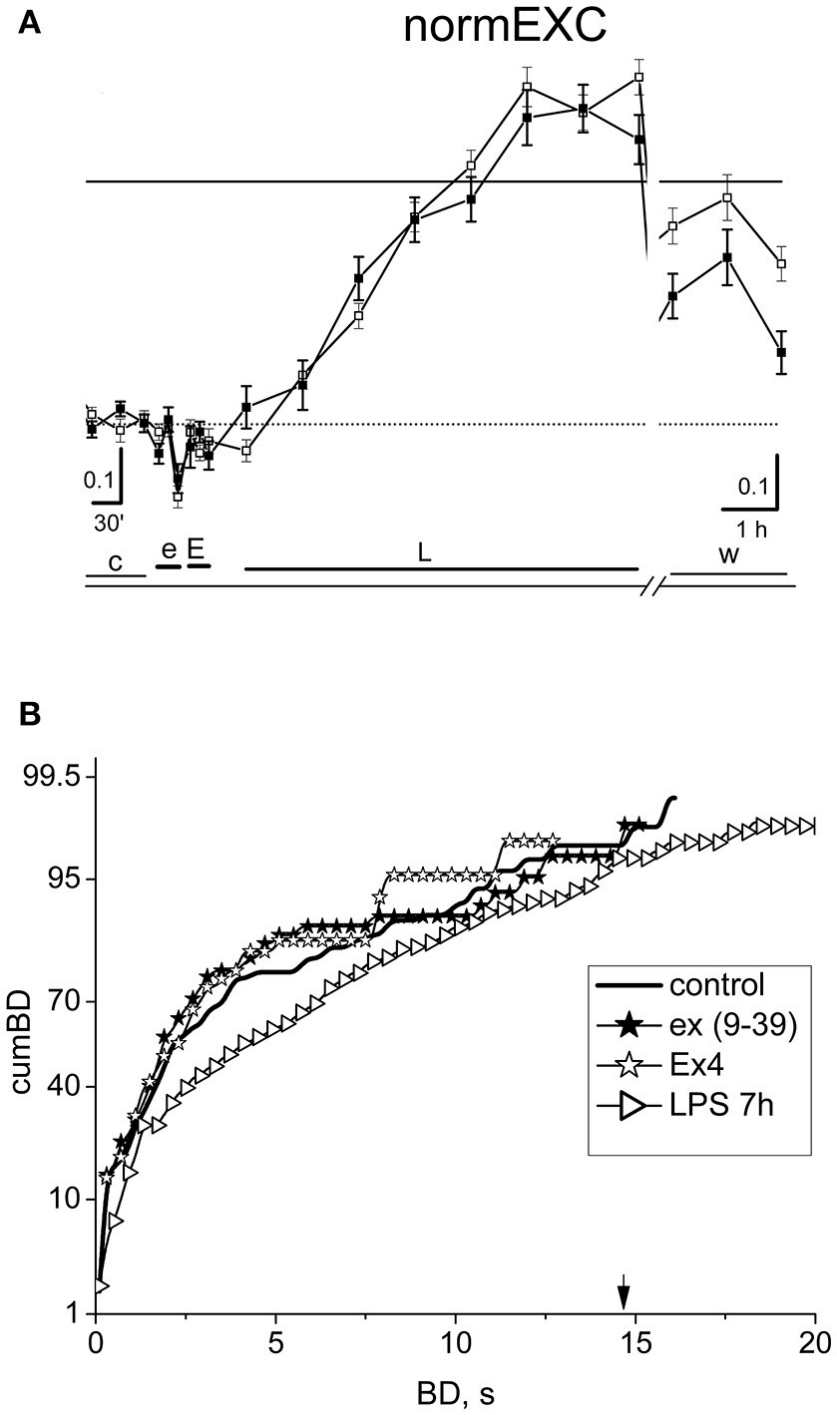
The effect of the specific antagonist of EX-4, exendin (9-39). **(A)** Plot of the normEXC in an experiment in which 100 nM exendin (9–39) (e) was applied for 30 min before 50 nM EX-4 (30 min, E) and the final long-term application of LPS (3 μg/ml). The continuous line is the fixed threshold. **(B)** Plot of cumBD in control (thick line), in the presence of exendin (9–39) (closed asterisk), or EX-4 added on top (open asterisk) and in LPS after 7 h (rightward triangles). The vertical arrow indicates the fixed threshold. The four curves were tested with the non-parametric Wilcoxon signed rank test analysis (exendin (9-39) vs. control, *P* < 10^−5^; LPS 7 h vs. control, *P* < 10^−10^; EX4 vs. exendin (9–39), *P* = 0.32). The GLP1-R antagonist blocked the protective effect of EX-4 observed in Figure 5, allowing the late excitatory responses to LPS to be expressed.

In order to confirm that the EX-4 action shown in Figure 5 is really due to the activation of GLP-1Rs, we performed experiments (*n* = 6) in which we preconditioned the EX-4 application (50 nM) with the specific GLP-1R antagonist exendin (9–39) at 100 nM. The results of such an exemplary experiment are shown in Figure 6. In Figure 6A, the normEXC plot shows that the early application of exendin (9–39) (e) induced a very short (10 min) and small negative deflection, followed by the application of EX-4 (E), and of LPS, which increased the slow normEXC up to 57 ± 3%. After 7 h, the activity stopped increasing. In Figure 6B the cumBD plots show that the 90% value was 8.1 and 12.2 s, in control and LPS, respectively, thus suggesting that a normal excitatory action of LPS took place as if EX-4 was not present. On the whole, these experiments confirm that the results shown in Figure 5 robustly indicate EX-4 as a potent agent to prevent LPS-induced inflammation.

### The levels of TNF-α, measured at 6 and 12 h after preconditioning with the polyphenols or EX-4 in LPS-treated dishes, is negligible compared to the levels found in non-preconditioned dishes

**Figure 7:**
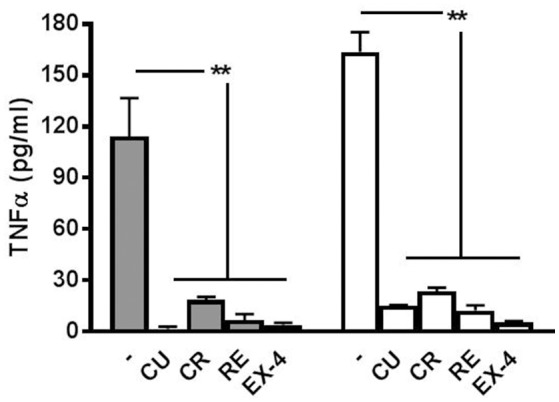
Release of TNF-α at 6 and 12 h after LPS application. The cytokine TNF-α is released from LPS-treated networks but not from networks pre-treated with polyphenols or exendin-4. Graph shows TNF-α concentration 6 h (filled bars) and 12 h (open bars) post-LPS (3 μg/ml) treatment with or without curcumin (CU, 1 μM) crocin (CR, 20 μM), resveratrol (RE, 200 nM) or exendin-4 (EX-4, 30 nM) pre-conditioning. Data are expressed as pg/ml and represent mean ± SEM of three independent experiments. ^**^P < 0.01 (ANOVA, Dunnet's test).

As reported in our previous paper (Gullo et al., 2014) where we showed that minocycline blocked the microglial-release of TNF-α induced by LPS treatment, we performed, for all the polyphenols and EX-4, appropriate experiments to test if the drug concentrations used in the experiments (Figures 2–5) were able to block also TNF-α production. As shown in Figure 7, the amount of this cytokine was, both at 6 and 12 h after LPS treatment, significantly smaller than that found when the preconditioning drugs were absent. On the whole, these results confirm that the drugs used here were not only able to block the atypical seizures induced by LPS, but also the release of TNF-α in the network dishes.

## Discussion

The present study is the first to demonstrate that polyphenols and the incretin GLP-1 hormone receptor agonist exendin-4 can mimic the anti-inflammatory action of minocycline (Yrjänheikki et al., 1998). In particular, they were able to block the seizure-like atypical activity caused by LPS and the concurrent TNF-α release from microglia, triggered by the Toll-like receptor 4 (TRL4) pathway, in a cortical co-culture of neurons, astrocytes, and microglia. The similar hyperactivity caused by LPS incubation was also observed with the application of a very low (60 pM) TNF-α concentration for ~12 h (Gullo et al., 2014), probably because of a marked change in the expression of glutamate transporters in astrocytes and microglia, as previously described (Persson et al., 2005; O'Shea et al., 2006; Takaki et al., 2012). The putative role of altered glutamate uptake in mediating components of the sterile inflammation response is currently under investigation in our cortical co-colture system (EW, FG, and ML), which includes functional astocytes (Wanke et al., 2016).

### Roles of microglia

Microglia are highly ramified cells which rapidly scan the local environment and react to its modification (Davalos et al., 2005). Under physiological conditions, microglia react rapidly to neuronal activity by modulating the contacts that their processes continuously establish with synaptic elements (Wake et al., 2009). Amongst the microglial-released molecules with a putative role in neurotransmission, TNF-α was shown to control basal synaptic functions (Santello et al., 2011) as well as plasticity (Stellwagen and Malenka, 2006; Costello et al., 2011), even if this role was attributed to TNF-α produced by astrocytes. However, the astrocytes have often been thought to release TNF-α because cultures of astrocytes are consistently contaminated by microglia (Saura, 2007). In fact, other data reveal no TNFα-encoding transcript in astrocytes (Foo et al., 2011; Zamanian et al., 2012). Thus, the TNF-α that controls several aspects of synaptic transmission is produced by microglial cells. Similarly, TLR4, the specific LPS receptor, was detected in mixed astrocyte/neuronal cultures, but when microglia were removed from astrocyte cultures, TLR4 expression was no longer present (Lehnardt et al., 2002).

### Polyphenols and their features of action

Apart from the obvious concentration-dependence, the effects of polyphenols can be subdivided into various types of action according to: (i) time-dependence (fast or long-term), (ii) cell-dependence (nervous or non-nervous system), and (iii) disease-dependence (i.e., Parkinson's, Alzheimer's diseases, or cancer). CU was found to be neuroprotective in the MPTP model of Parkinson's disease (Ojha et al., 2012) by inhibiting the generation of pro-inflammatory cytokines through the prevention of NF-κB traslocation into the nucleus (Karunaweera et al., 2015). In addition, CU attenuated inflammation in experimental traumatic brain injury (Zhu et al., 2014) and decreased human TNF-α levels (Sahebkara et al., 2016). The nanoencapsulated CU version was also found to reduce β-amyloid-induced cognitive impairment in rats (Hoppe et al., 2013). In the same way as CU, RE was also able to protect dopamine neurons (Zhang et al., 2010) from LPS-induced inflammation. Moreover, RE was found to protect cortical neurons from oxygen-glucose deprivation-induced apoptosis (Gao et al., 2014). In general, RE studies in *in vitro* and *in vivo* showed interesting data of metabolism and bioavailability in various animal models and in humans (Smoliga and Blanchard, 2014; Gambini et al., 2015). Interestingly, RE was shown to ameliorate the clinical severity observed in an animal model of multiple sclerosis, by maintaining the integrity of the blood-brain barrier (Wang et al., 2016). In summary, we believe that the principal mechanisms by which polyphenols were able to protect against LPS neuroinflammation in our system are those related to the TLR4 pathway and microglia activation.

### The action of GLP-1 in the CNS

There is evidence that the gut hormone GLP-1 is also produced in the brain (Korol et al., 2015; Thiebaud et al., 2016). In particular, it was shown that the GLP-1 receptor agonist exendin-4 (EX-4) protected dopaminergic neurons by inhibiting TNF-α release from microglia (Kim et al., 2009). A reduction of brain TNF-α levels was also described in an animal model of Alzheimer disease (AD) exposed to EX-4 treatment (Solmaz et al., 2015). In LPS-treated rats, GLP-1 protected hippocampal neurons from synaptic impairments (Iwai et al., 2014) and inhibited IL-1β production from cultured astrocytes (Iwai et al., 2006). Furthermore, *in vitro* administration of EX-4 in rats increased the expression of the astrocytic glutamate transporter GLT-1 in the hippocampus, lowering extracellular glutamate concentration (Kobayashi et al., 2013). This is in line with the lower long-term excitability data shown in Figures 5B,C during LPS inflammation. The results shown in Figures 5, 6 split the presumed fast AMPAR-induced transient firing increase following GLP-1R activation (by EX-4), as characterized by Oka et al. (1999), from the long-term effects related to the switch-off of the LPS-induced inflammation.

Our data show, quite importantly, and for the first time, that these effects are not peculiar to the hippocampus *in vivo*, but can also be reproduced *in vitro*, in networks from neocortical dissociated cells containing neurons, astrocytes, and microglia. Moreover, the data shown in Figure 6 demonstrate that the GLP-1R antagonist exendin (9–39) functionally blocked the ability of EX-4 to counteract the LPS sterile inflammation. Thus in summary, we think that the intriguing connection between the fast electrophysiological responses and the slow microglia pathway merits a specialized investigation, difficult to be carried out by means of our procedures.

### Ion channels in microglia

Only recently, ion channels have also been described to be functionally expressed in microglia (for a minireview, see Madry and Attwell, 2015). Among the various types of identified channels, the voltage-gated proton channel Hv1 (not present in neurons) was thought to have a dramatic importance during damage induced by ischemic stroke when reactive oxygen species (ROS) are generated (Wu et al., 2012). Accordingly, knock-out mice lacking the Hv1 channel were protected from ROS-mediated neuronal death and brain damage. Furthermore, since acute neuroinflammation provokes intracellular acidification (Tyrtyshnaia et al., 2016), it cannot be excluded that voltage-gated proton channels or acid-sensing channels (Yu et al., 2015) could also be involved in this type of process. Indeed, a polyphenol such as epigallocatechin-3-gallate (EGCG) is known to inhibit the Hv1 currents in microglial BV2 cells (Jin et al., 2012). Interestingly, the reduction in TNF-α release produced by some antidepressants was supposed to be related to the same effect (Song et al., 2012). The putative role of this ionic mechanism in the LPS-induced sterile inflammation is also currently under investigation in our laboratory (EW, FG, and ML).

## Author contributions

AC, ML, BC, and EW conceived and designed the research; FG, MC, and AD performed the experiments; EW and BC analyzed the data; EW and AC drafted the manuscript.

### Conflict of interest statement

The authors declare that the research was conducted in the absence of any commercial or financial relationships that could be construed as a potential conflict of interest.

## Supplementary material

Supplementary Figure 1 Dose-response relationship for crocin (CR) and exendin-4 (EX4). Normalized excitability (normEXC) is represented for excitatory and inhibitory neurons.
